# A survey investigation of the imaging practices of human body donors in gross anatomy laboratories

**DOI:** 10.1002/ase.70339

**Published:** 2026-08-20

**Authors:** Danielle N. Edwards, Rhiannon E. Robinson, Bobbie J. Leeper, Kelsey E. Stevens, Jeremy J. Grachan

**Affiliations:** ^1^ Department of Cell, Developmental, and Integrative Biology University of Alabama at Birmingham Heersink School of Medicine Birmingham Alabama USA; ^2^ Department of Anatomy and Neurobiology Boston University Aram V. Chobanian & Edward Avedisian School of Medicine Boston Massachusetts USA; ^3^ Biology and Physician Assistant Programs Seton Hill University Greensburg Pennsylvania USA; ^4^ Exercise and Health Sciences Department Briar Cliff University Sioux City Iowa USA; ^5^ Department of Medicine Rutgers New Jersey Medical School Newark New Jersey USA; ^6^ Present address: University of Louisville, School of Medicine Louisville Kentucky USA

**Keywords:** cadavers, gross anatomy laboratory, human body donors, multimedia, photography, video

## Abstract

The imaging of human body donors, as photographs and videos, is increasing in need as digital access of educational materials increases. In the past several years, several professional societies have published guidelines and recommendations for imaging human body donors for all who work with human body donors. However, the implementation of policies, the storage of images, and the compliance with these policies are largely unknown. The Human Anatomy and Physiology Society Anatomical Donor Stewardship Committee distributed a survey to determine the policies for imaging human body donors to institutions that use human body donors through professional anatomy organization listservs. A total of 74 (*n* = 74) responses were returned. The most common policy was to request permission before any imaging of human donors. Imaging policies were influenced by the amount of donors, where the donors were obtained (internal programs, external programs, or mixed), but not by the type of institution or the use of the donors (dissection, prosection, etc.). Of those who took images, photographs were more common over videos for both educational and research use. However, those who took videos for education mainly reported policies prohibiting imaging. When storing images, more restrictive imaging policies were more likely to report secure storage of digital donor materials. This study shows that each institution still has a variety of policies for imaging of human donors, and not all follow the published guidelines. Overall, the landscape of current policies demonstrates a strong need to standardize policies for imaging of donors.

## INTRODUCTION

Human body donor dissection and donor prosection for the purpose of anatomy education have served as the gold standard pedagogical method by offering intimate, three‐dimensional, and relational exploration of human structures with their real complexity and variation.^1^ Furthermore, work with human anatomy donors is often credited with fostering professional identity formation (e.g., respect for the donor, initial patient care, and confronting mortality).^2^ Due to its impact, many curricula retain dissection as a core component, despite the additional time, expense, and controlled access required to maintain such a program.

While dissection remains at the forefront of many health care profession curricula, the landscape of anatomy education is shifting. Reductions in contact hours, resource constraints, safety concerns (e.g., formaldehyde exposure), aging infrastructure requiring expensive renovation, and significant curricular modifications have led programs to re‐examine and/or reduce dissection‐based hours or rely more heavily on prosected tissues, models, and digital resources.^3^ The evolving pre‐clinical curricula, in addition to the changes brought on by the COVID‐19 pandemic, have both increased expectations and requirements for digital study materials from both students and accreditation bodies.^4^, ^5^

This digital shift alters the landscape of modern anatomy education. Previous atlases involved hand‐drawn illustrations and date back to Vesalius's atlas in 1543,^6^ but contemporary atlases often show real human tissue of human body donors, such as the *Color Atlas of Anatomy: A Photographic Study of the Human Body*.^7^ Outside of published atlases, there is an increased demand for institutional and program‐specific material like dissectors, atlases, etc. resulting in a demand for “real” tissue‐based anatomy that can be accessed and disseminated digitally. While at the surface this idea seems to be a natural progression with current educational advances, the shift to digital access of human tissue from human body donors raises significant ethical concerns. With these educational changes comes the need for ethical guidelines and policies to protect the trust, privacy, and security of the donor and their families.

Digital images pose a particularly critical ethical issue as they are potentially immortal and ungovernable depictions of the donor's body that can be stored indefinitely and are easily shared. Images of the deceased have the power to influence society's views on dissection, and misuse of the images can damage the trust and relationship between body donors, donor families, and the donor programs, possibly affecting the willingness for individuals to donate.^8^ Additionally, taking an image of a person without their consent violates basic societal standards of decency, and is an ethical breach of conduct (at minimum) for any medical‐ or medical‐adjacent setting.^9^ Despite these issues, there has been a lack of guidelines or regulations until recently. Leeper et al.^10^ reviewed 15 published ethical guidelines for the treatment of human body donors, but only six of them specifically mention limiting or prohibiting images of the donors. However, each guideline focused on different ethical concerns with imaging. This includes limiting access of images such as prohibiting posting of images to the public domain (websites, social media, etc.) to reduce “morbid curiosity”^11^, ^12^ and limiting images to education or research exclusively.^13^, ^14^, ^15^ A common theme was the need for consent from donors for imaging their remains, treating the donors with the same respect and privacy afforded through laws, such as HIPPA.^11^, ^14^, ^16^

Since 2012, three societies, the American Association of Clinical Anatomists (AACA), the American Association for Anatomy (AAA), and the International Federation of Associations of Anatomists (IFAA), have each published guidelines regarding imaging of human body donors, though the extent of recommendations varied. The best practices guidelines from AACA list a single suggestion within their “Best Practices for Donor Programs” on the detailed language used in agreements for the use of anatomical materials and intended use, including any images.^17^ The AAA's best practices guideline has a section dedicated to imaging of human body donors. Specifically, it centered discussions on potential legal and program repercussions with the dissemination of any donor image to the public, with and without identifiable features. The AAA's suggestions ultimately focused on emphasizing oversight by the body donor program, the storage of images on secure/encrypted servers and not personal devices, and limited access to those images.^18^ The IFAA has published two guidelines in 2012 and 2023, with the 2023 guidelines being solely dedicated to imaging of human body donors, and as such, is the most comprehensive.^11^, ^19^ These guidelines expanded on the previous guidelines set by the AACA, AAA, and IFAA with additional recommendations encouraging explicit donor consent, limiting use as any other anatomical material, avoiding taking images with identifiable features, regardless of use, the secure storage of images, prohibiting commercialization of donor images, disclosing sources for any dataset, and destroying images that are not being used for educational or research use. Collectively, these documents show an increase in considerations with the use of human body donor images.

Despite these advances, opportunities, and challenges, there is a lack of systematic data on the guidelines that anatomists use. Such knowledge can help the anatomy education community to identify opportunities for sharing (e.g., the donor institution provides specific images other institutions can use), provide practical guidance regarding benchmark best‐practices and standardization, insight into how to better draft consent forms to meet their needs, and potentially toward fostering infrastructural or policy development. To address this gap, the Human Anatomy and Physiology Society's (HAPS) Anatomical Donor Stewardship (ADS) Committee conducted a survey of anatomists to explore the policies, prevalence, storage methods, and viewpoints around human anatomy donor imaging in anatomical education. This study aimed to present the landscape of how and why anatomists capture anatomy donor images, analyze associations between institutional and pedagogical characteristics and imaging practice, and discuss implications for future educational development, ethics, and resource sharing in the anatomical sciences. Finally, the HAPS ADS provides suggestions toward best practices in anatomy imaging.

## METHODS

### Survey

During the Fall of 2024 and Spring of 2025, a survey was distributed to members of the Human Anatomy and Physiological Society (HAPS) via the HAPS Discussion Group listserv and to members of AAA via Anatomy Connected (listserv). Members were also encouraged to forward the document to colleagues outside of the organization. The survey was anonymous and was accessed and completed through Qualtrics (Qualtrics, Provo, UT) through a single link. Both HAPS and AAA have members nationally and internationally; however, the exact country was not surveyed in this study. The survey consisted of 20 close‐ended and dichotomous (yes/no) questions designed to ascertain participant demographics, gross laboratory experience, imaging practices, and imaging storage questions. The questions were developed using the Delphi process^20^ by members of the HAPS ADS Committee (*n* = 5, the co‐authors), who have previous experience in survey design and are experts in gross anatomy laboratory practices. Drafts of the survey were then reviewed and evaluated by other anatomists (*n* = 5), whose feedback was integrated into the final version of the survey.

### Inclusion and exclusion criteria

Participants who work in a gross anatomy laboratory with donors were included in the analysis. Those who did not use human body donors (*n* = 9) were excluded. Additionally, those who did not respond to the first questions (*n* = 7), had a duplicate IP address (*n* = 3), or did not consent to the study (*n* = 1) were removed.

### Statistical analysis

All survey responses were exported from Qualtrics, cleaned, and analyzed within Microsoft Excel (Microsoft Corporation, Redmond, WA). Responses were categorized into similar groups and percentages focusing on group themes were calculated. Chi‐squared analysis and Fischer's exact test were performed to determine significance among different groups. Fischer's exact test was chosen when more than 20% of categories had less than five occurrences.^21^ Chi‐squared analysis and Fischer's exact test were performed on number of occurrences, not percentages. Results were deemed statistically significant with *p*‐values of *p* < 0.05. Figures and statistical analysis were performed with GraphPad Prism Version 9 (GraphPad Software, Boston, MA).

### Ethics review

This study was given exempt status by the University of Alabama at Birmingham's Institutional Review Board (IRB‐300012853).

## RESULTS

### Demographics

Out of 94 surveys, 74 (78.7%) surveys met inclusion criteria for the study. Most participants reported working in a 4‐year public institution (*n* = 22, 29.7%), a 4‐year private institution (*n* = 21, 28.4%), or a community college (*n* = 14, 18.9). There was an equal distribution in the size of the institution with 31.1% (*n* = 23) enrolling less than 5000 students, 32.4% (*n* = 24) enrolling between 5000 and 15,000, and 36.5% (*n* = 27) enrolling more than 15,000 students. Additional institutional demographics can be found in Table 1.

**TABLE 1:** Institutional demographics (*n* = 74).

	Number (*n*)	Percent (%)
*Type of institution*		
Four‐year public institution	22	29.7
Four‐year private institution	21	28.4
Community college	14	18.9
Two‐year public institution	1	1.4
Graduate/profession programs only	9	12.2
For‐profit	1	1.4
Other	6	8.1
*Size of institution*		
Less than 5000 students	23	31.1
Between 5000 and 15,000 students	24	32.4
More than 15,000 students	27	36.5

### Gross anatomy laboratory program characteristics

For most gross anatomy laboratories, human body donors were provided solely by outside of programs/organizations, programs outside of their institution who supply their human body donors (*n* = 45, 60.8%), followed by donors provided solely from internal programs (*n* = 16, 21.6%). Within an academic year, the number of donors was evenly distributed between small programs (0–5 donors, *n* = 23, 31.1%), medium programs (6–40 donors, *n* = 28, 37.8%), and large programs (41+ donors, *n* = 19, 25.7%). These donors were most commonly kept for one (*n* = 35, 47.3%) to two (*n* = 17, 23.0%) years and were fully embalmed (*n* = 71, 95.9%) or fresh/unembalmed (*n* = 17, 23.0%). Human body donors were mostly used by undergraduate students (*n* = 63, 85.1%) and professional students (*n* = 43, 58.1%) through use in dissection (*n* = 63, 85.1%), prosection (65, 87.8%), and outreach programs (*n* = 40, 54.1%). Additional information about human body donor programs and their use can be found in Table 2.

**TABLE 2:** Institutional gross anatomy laboratories demographics (*n* = 74).

	Number (*n*)	Percent (%)
*Supplier of human body donors*		
Internal donor program	16	21.6
Donors from other/larger organizations	45	60.8
Both internal donor program and donors from larger/other organizations	5	6.8
Other	8	10.8
*Number of donors per academic year*		
0–5	23	31.1
6–15	20	27.0
16–40	8	10.8
41–100	12	16.2
100+	7	9.5
Other	3	4.1
*Length of donor use*		
1 semester	6	8.1
1 year	35	47.3
2 years	17	23.0
3 years	7	9.5
4 years	3	4.1
5–9 years	2	2.7
10+ years	3	4.1
*Type of donor preservation*		
Fully embalmed	71	95.9
Partially embalmed	9	12.2
Fresh (unembalmed)	17	23.0
Frozen (unembalmed)	9	12.2
Other	1	1.4
*Type of donor use*		
Prosection	65	87.8
Dissection	63	85.1
Outreach	40	54.1
Continuing education	33	44.6
Surgical Training	22	29.7
Research	21	28.4
*Students utilizing donors*		
High school groups	35	47.3
Undergraduate students	63	85.1
Graduate students	35	47.3
Professional students	43	58.1

*Note*: The questions for supplier of human body donors, type of donor preservation, type of donor use, and students utilizing donors were all select all that apply options.

### Imaging policies

Regarding policies within the gross anatomy laboratory, both the type of allowed images (photographs or videos) and the types of policies that regulated their use were investigated. The predominant regulatory policies for imaging of human body donors was for the donor custodian to obtain permission to take photographs/images and follow regulations set by the donor program (*n* = 36, 48.6%), followed by the prohibition of taking images (*n* = 14, 18.9%), and lastly the policy that photographs could be taken without permission as long as regulations were followed (e.g., no exposed faces or identifiable tattoos) (*n* = 12, 16.2%). Survey results found that photographs (*n* = 35, 47.3%) and videos (*n* = 50, 67.6%) were not commonly being taken of human body donors. When used, both photographs (*n* = 29, 39.2%) and videos (*n* = 22, 29.7%) were more likely to be used for educational purposes rather than research purposes. For those who took images of donors (*n* = 36), storage of photographs and videos on a work computer (*n* = 30, 83.3%) or secure/encrypted server (*n* = 23, 63.9%) were the most common, followed by cloud storage (*n* = 16, 44.4%). Of note, the use of personal phones (*n* = 8, 22.2%) and personal computers (*n* = 6, 16.7%) were common places of image storage. Additional information on reported imaging policies and storage can be found in Table 3.

**TABLE 3:** Imaging policies in gross anatomy laboratories (*n* = 74).

	Number (*n*)	Percent (%)
*Do you take photographs of the donors?*		
Yes, to use images in class for educational purposes	29	39.2
Yes, to use for research	20	27.0
No	35	47.3
Other	6	8.1
*Do you take videos of the donors?*		
Yes, for my class to watch for educational purposes	22	29.7
Yes, to use for research	4	5.4
No	50	67.6
Other	2	2.7
*What are the regulations that you follow for taking photographs/videos of the donors?*		
I am able to take any photographs/videos I want; there are no limitations	5	6.8
I am able to take photographs/videos without permission, but with certain regulations (e.g., no exposed faces or identifiable tattoos)	12	16.2
I have to request permission to take photographs/videos and follow regulations set by the donor program	36	48.6
I am not allowed to take photographs/videos of donors	14	18.9
I do not know whether there are regulations for taking photographs/videos of the donors	1	1.4
Other	4	5.4
*If you do not take photographs/videos of the donors, please explain why*.		
I am not allowed to take photographs/videos of the donors	14	77.8
I have no need to take photographs/videos	11	61.1
Ethical issues	7	38.9
I do not know what the regulations are for taking photographs/videos of donors	3	16.7
*If you take photographs/videos of donors, where do you store the media?*		
Work computer	30	83.3
Secure/Encrypted drive/server	23	63.9
Cloud space	16	44.4
Personal phone	8	22.2
Personal computer/laptop	6	16.7

*Note*: The question “If you take photo/videos of donors, where do you store the media?” is an *n* = 36. All questions are select all that apply.

For those who do not take photographs and/or videos (*n* = 18) from policies (*n* = 14) or personal viewpoints (*n* = 3), the most common reported reasons were they were not allowed (*n* = 14, 77.8%), had no need to take photographs/videos (*n* = 11, 61.1%), or there were ethical issues with taking photographs/videos (*n* = 7, 38.9%). More information is detailed in Table 3.

### Factors influencing imaging policies

When comparing the size of the donor program against the type of donor imaging policies, there was a significant difference (*p* = 0.0048) between small (0–5 donors), medium (6–40 donors), and large (41+ donors) programs on the type of imaging policies that were used (excluding “I do not know” and “Other” responses). Interestingly, the difference was more significant when small donor programs were compared with medium programs (*p* = 0.0485) and large programs (*p* = 0.0016) (Figure 1). This significant difference was not found when medium and large donor programs were compared (*p* = 0.0980). Additional comparisons between imaging policies and type of institution (e.g., 4‐year public and 4‐year private) (Figure 2) and imaging policies and donor use (e.g., prosection and dissection) (Figure 3) did not yield any significant differences (*p* = 0.7790, *p* = 0.7761, respectively).

**FIGURE 1:**
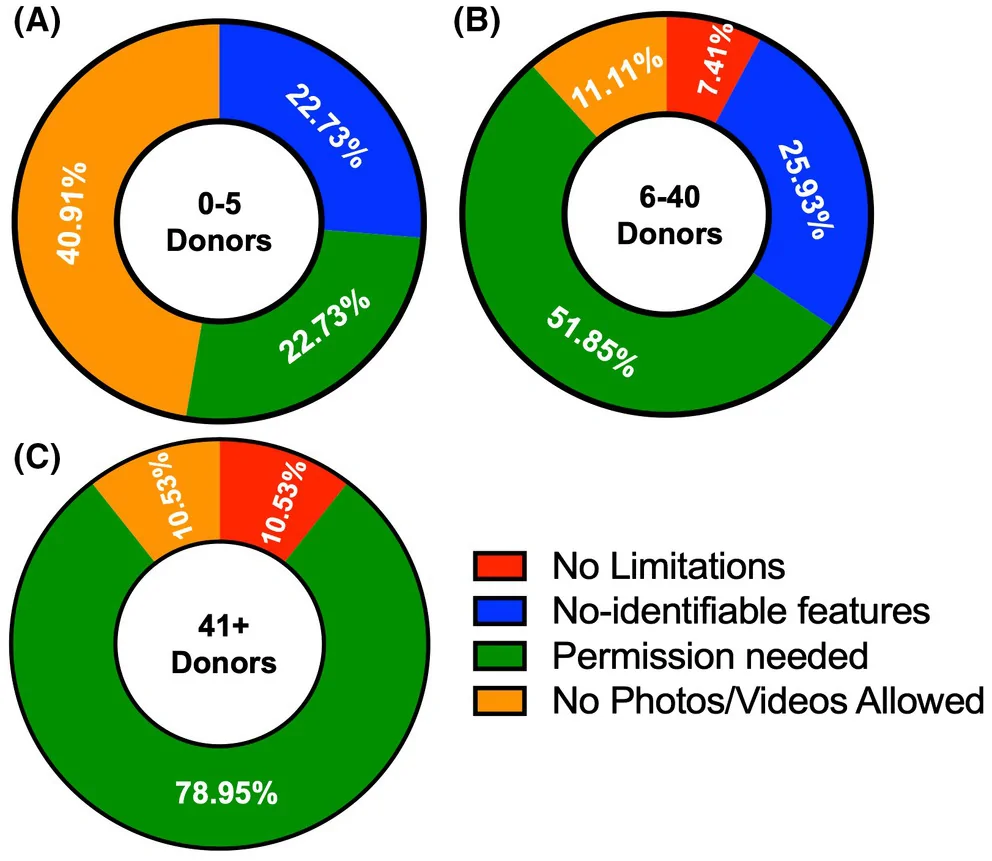
Chi‐squared analysis of imaging policies and human body donor program size at responding institutions. (A) Small (*n* = 23): No limitations 0% (*n* = 0), No‐identifiable features 22.73% (*n* = 5), Permission needed 22.73% (*n* = 5), No photographs/videos allowed 40.91% (*n* = 9). (B) Medium (*n* = 28): No limitations 7.41% (*n* = 2), No‐identifiable features 25.93% (*n* = 7), Permission needed 51.85% (*n* = 14), No photographs/videos allowed 11.11% (*n* = 3). (C) Large (*n* = 19): No limitations 10.53% (*n* = 2), No‐identifiable features 0% (*n* = 0), Permission needed 78.95% (*n* = 15), No photographs/videos allowed 10.53% (*n* = 2). Responses of “I do not know,” “Other,” and blanks were removed from analysis. No Limitations = “I am able to take any photos/videos I want; there are no limitations”; No‐identifiable features = “I am able to take photos/videos without permission, but with certain regulations (e.g., no exposed faces or identifiable tattoos)”; Permission needed = “I have to request permission to take photos/videos and follow regulations set by the donor program”; and No Photographs/Videos Allowed = “I am not allowed to take photos/videos of donors.” Small (*n* = 23) vs. medium (*n* = 28) donor programs Chi‐square p* = 0.0485. Small (*n* = 23) vs. large (*n* = 19) donor programs Chi‐square p** = 0.0016. Medium (*n* = 28) vs. large (*n* = 19) donor programs Chi‐square *p*(NS) = 0.0980). Donor program size vs. imaging policies Chi‐square p** = 0.0048.

**FIGURE 2:**
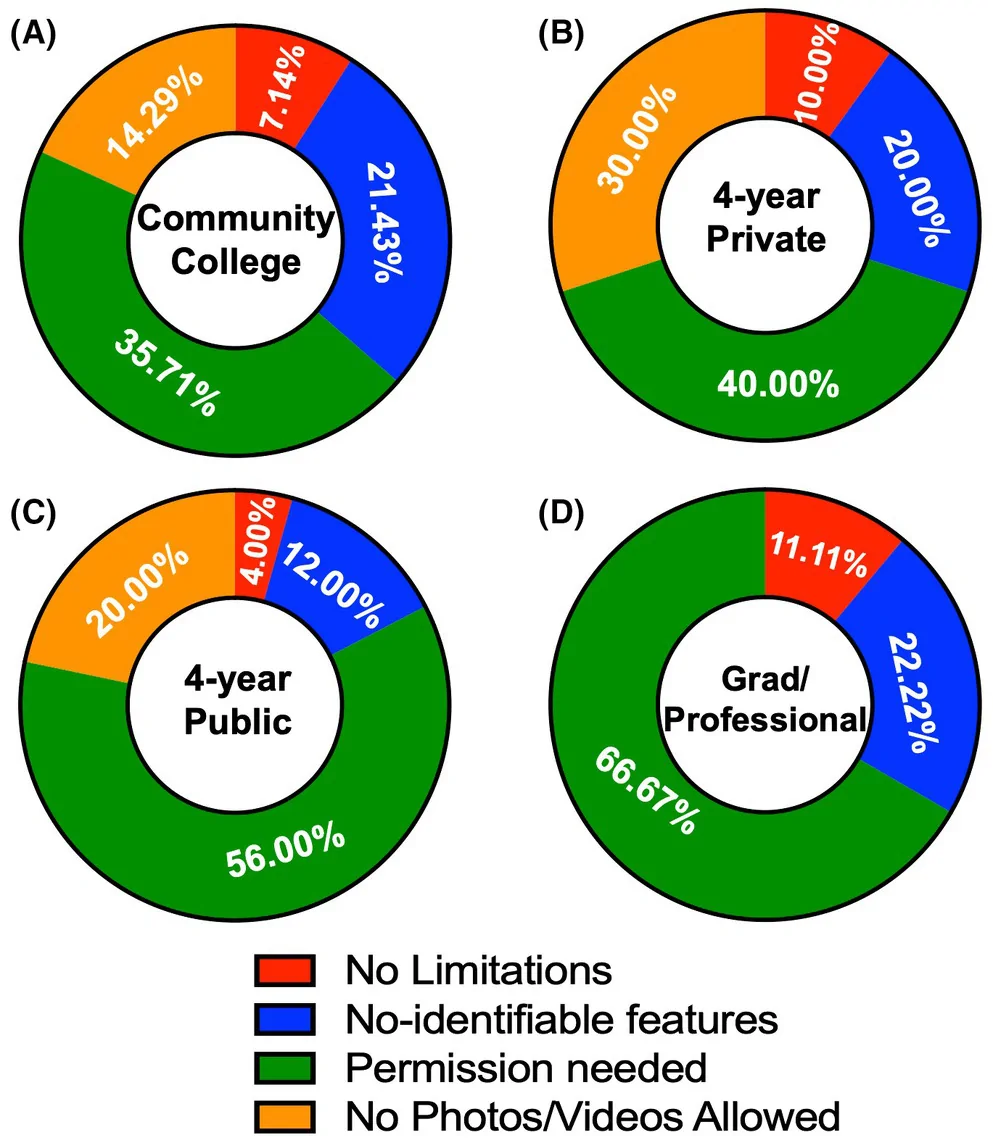
Chi‐squared analysis of imaging policies and type of institution. (A) Community College (*n* = 14): No limitations 7.14% (*n* = 1), No‐identifiable features 21.43% (*n* = 3), Permission needed 35.71% (*n* = 5), No photographs/videos allowed 14.29% (*n* = 2). (B) Four‐year Private Institution (*n* = 20): No limitations 10.00% (*n* = 2), No‐identifiable features 20.00% (*n* = 4), Permission needed 40.00% (*n* = 5), No photographs/videos allowed 30.00% (*n* = 2). (C) Four‐year Public Institution (*n* = 25): No limitations 4.00% (*n* = 1), No‐identifiable features 12.00% (*n* = 3), Permission needed 56.00% (*n* = 14), No photographs/videos allowed 20.00% (*n* = 5). (D) Graduate/Professional Only Institution (*n* = 9): No limitations 11.11% (*n* = 1), No‐identifiable features 22.22% (*n* = 2), Permission needed 66.67% (*n* = 6), No photographs/videos allowed 0.00% (*n* = 0). Responses of “I do not know,” “Other,” and blanks were removed from analysis. No Limitations = “I am able to take any photos/videos I want; there are no limitations”; No‐identifiable features = “I am able to take photos/videos without permission, but with certain regulations (e.g., no exposed faces or identifiable tattoos)”; Permission needed = “I have to request permission to take photos/videos and follow regulations set by the donor program”; and No Photographs/Videos Allowed = “I am not allowed to take photos/videos of donors.” Chi‐square analysis *p*(NS) = 0.7790.

**FIGURE 3:**
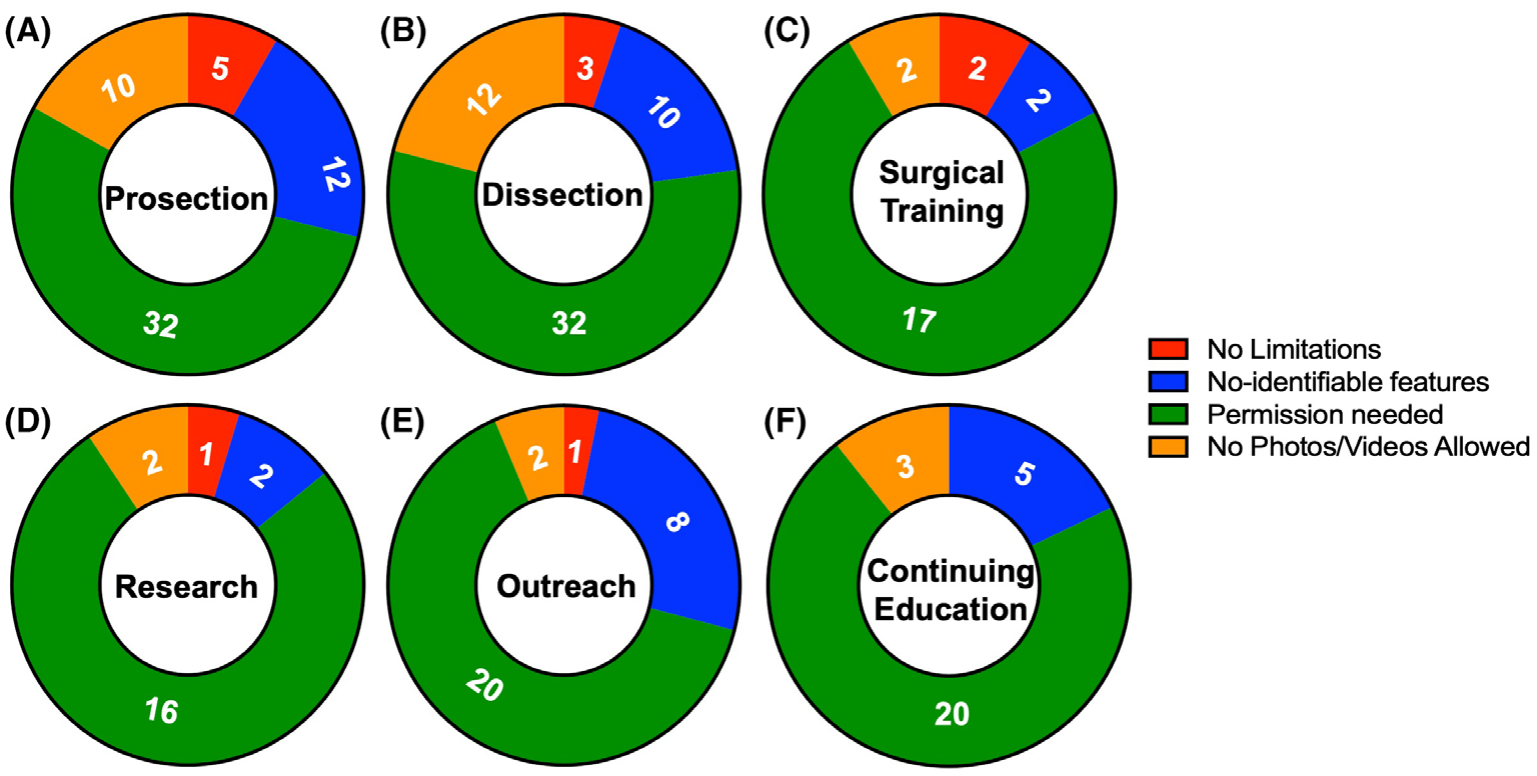
Chi‐squared analysis of imaging policies and in‐lab donor use. (A) Prosection: No limitations *n* = 5, No‐identifiable features *n* = 12, Permission needed *n* = 32, No photographs/videos allowed *n* = 10. (B) Dissection: No limitations *n* = 3, No‐identifiable features *n* = 10, Permission needed *n* = 32, No photographs/videos allowed *n* = 12. (C) Surgical Training: No limitations *n* = 2, No‐identifiable features *n* = 2, Permission needed *n* = 17, No photographs/videos allowed *n* = 2. (D) Research: No limitations *n* = 1, No‐identifiable features *n* = 2, Permission needed *n* = 16, No photographs/videos allowed *n* = 2. Responses of “I do not know,” “Other,” and blanks were removed from analysis. (E) Outreach: No limitations *n* = 1, No‐identifiable features *n* = 8, Permission needed *n* = 20, No photographs/videos allowed *n* = 2. (F) Continuing Education: No limitations *n* = 0, No‐identifiable features *n* = 5, Permission needed *n* = 20, No photographs/videos allowed *n* = 3. This question was select all that apply, and the data are reported as number of occurrences. Responses of “I do not know,” “Other,” and blanks were removed from analysis. No Limitations = “I am able to take any photos/videos I want; there are no limitations”; No‐identifiable features = “I am able to take photos/videos without permission, but with certain regulations (e.g., no exposed faces or identifiable tattoos)”; Permission needed = “I have to request permission to take photos/videos and follow regulations set by the donor program”; and No Photographs/Videos Allowed = “I am not allowed to take photos/videos of donors.” Chi‐square analysis p* = 0.0485. Small (*n* = 23) vs. large (*n* = 19) donor programs Chi‐square p** = 0.0016. Medium (*n* = 28) vs. large (*n* = 19) donor programs Chi‐square *p*(NS) = 0.0980). Donor program size vs. imaging policies Chi‐square analysis *p*(NS) = 0.7761.

The type of donor program (i.e., internal body donor program, external body donor program, or combination of internal and external body donor programs) and the type of imaging policies for each of these donor programs was compared. When looking at internal donor programs (*n* = 15, Figure 4A), most policies required permission from the donor program to take images (*n* = 12, 80%), followed by no limitations on the images they could take (*n* = 2, 13.33%) and not being allowed to take images (*n* = 1, 6.67%). For those who reported external donor programs (*n* = 45, Figure 4B), again most policies required permission from the donor program to take images (*n* = 19, 42.22%). However, the second most common policy was no need to request permission as long as certain regulations were followed (*n* = 11, 24.44%). For those who use both internal and external donor programs (*n* = 5, Figure 4C), they overwhelmingly were allowed to take images only with permission from the program (*n* = 4, 80%). Fisher's exact test showed a significant difference (p* < 0.0001) between where body donors were obtained and the imaging policies.

**FIGURE 4:**
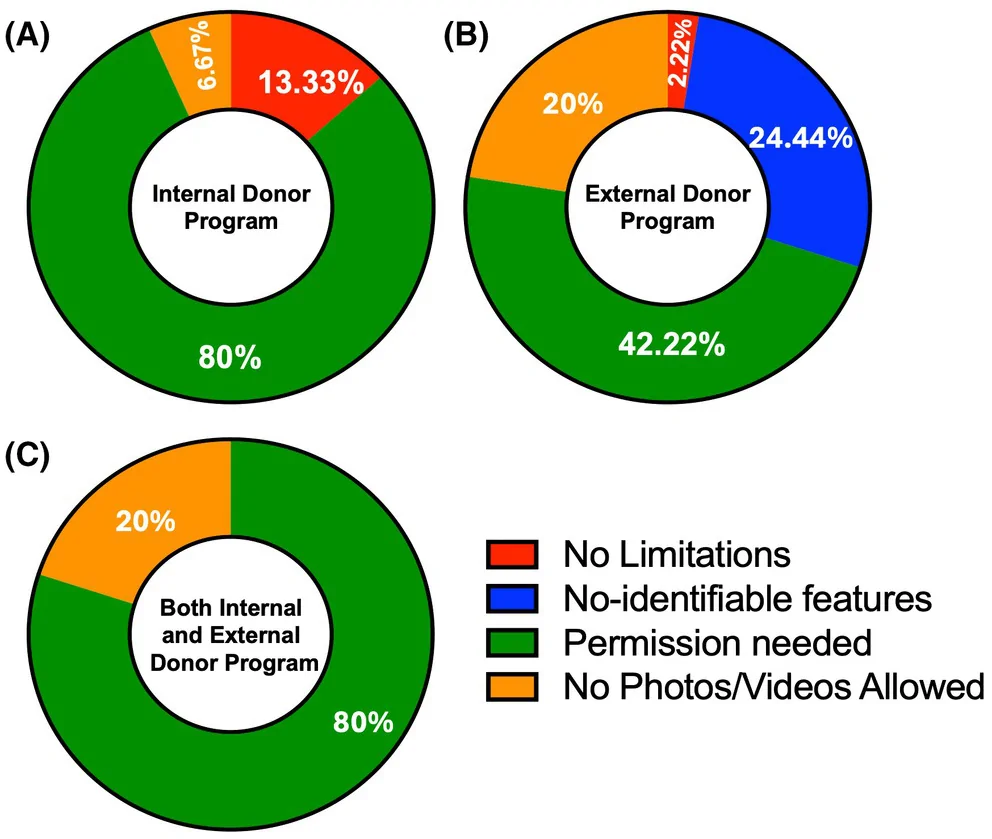
Percentage of the type of imaging policies within programs on obtaining body donors. (A) Internal donor programs (*n* = 15): No limitations 13.33% (*n* = 2), Regulated 0% (*n* = 0), Permission needed 80% (*n* = 12), No photographs allowed 6.67% (*n* = 1). (B) External donor program (*n* = 45): No limitations 2.22% (*n* = 1), No‐identifiable features 24.44% (*n* = 11), Permission needed 42.22% (*n* = 19), No photographs allowed 20% (*n* = 9). (C) Both internal and external donor programs (*n* = 5): No limitations 0% (*n* = 0), Regulated 0% (*n* = 0), Permission needed 80% (*n* = 4), No photographs/videos allowed 20% (*n* = 1). Responses of “I do not know,” “Other,” and blanks were removed from analysis. No Limitations = “I am able to take any photos/videos I want; there are no limitations”; No‐identifiable features = “I am able to take photos/videos without permission, but with certain regulations (e.g., no exposed faces or identifiable tattoos)”; Permission needed = “I have to request permission to take photos/videos and follow regulations set by the donor program”; and No photographs/videos allowed = “I am not allowed to take photos/videos of donors.” Fischer's exact test p**** < 0.0001.

To confirm the findings found in Figures 1 and 4, additional analysis was performed to determine the type of body donor program (i.e., internal body donor program, external body donor program, or combination of internal and external body donor programs) at an institution was related to the number of human body donors at an institution (small 0–5, medium 6–40, and large 41+). Fischer's exact test revealed a significant difference (*p* < 0.0001) between the different sized programs compared to where they obtained their donors. Large programs were more likely to obtain donors from internal programs (*n* = 10, 58.82%, Figure 5C), compared with small programs and medium programs. Institutions were more likely to obtain donors from external donor programs for small (*n* = 21, 91.30%, Figure 5A) and medium programs (*n* = 20, 76.92%, Figure 5B, respectively). This revealed that the smaller the program (<41), the more likely that the program obtains donors from an external program with a variety of policies. However, the larger the program (41+), the more likely the program obtains their donors from internal programs where the policies are much more uniform and require permission.

**FIGURE 5:**
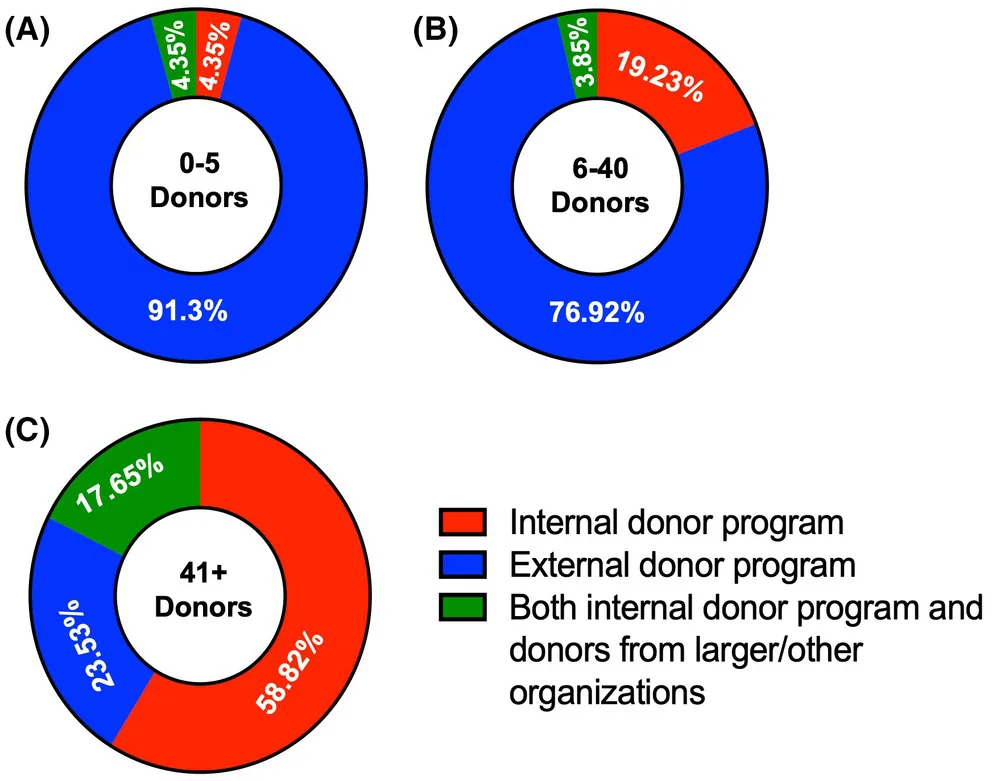
Fischer's exact test analysis on the size of the donor program and the type of program obtaining body donors. (A) Small programs (0–5 donors) (*n* = 23): Internal donor program (*n* = 1, 4.35%), External donor program (*n* = 21, 91.30%), Both internal donor program and donors from larger/other organizations (*n* = 1, 4.35%). (B) Medium programs (*n* = 26): Internal donor program (*n* = 5, 19.23%), External donor program (*n* = 20, 76.92%), Both internal donor program and donors from larger/other organizations (*n* = 1, 3.85%). (C) Large programs (*n* = 14): Internal donor program (*n* = 10, 58.82%), External donor program (*n* = 4, 23.53%), Both internal donor program and donors from larger/other organizations (*n* = 3, 17.65%). Responses of “Other” were removed. Fischer's exact test on number of occurrences *p* **** < 0.0001.

An analysis into how the type of imaging policies might influence the use of photographs and images (i.e., for education or research) was investigated next. For those who took photographs for educational use (*n* = 28, Figure 6A), they were most likely to have policies that required permission from the donor program (*n* = 17, 60.71%), followed by policies that did not require permission, but had to exclude identifiable features (*n* = 6, 21.43%) and policies that had no limitations (*n* = 3, 10.71%). Similarly to educational use, those who took photographs for research purposes (*n* = 20, Figure 6B) were most likely to have policies that required permission from the donor program (*n* = 15, 75.00%), followed by policies that required no‐identifiable features (*n* = 2, 10.00%) or had no limitations (*n* = 2, 10.00%). On the contrary, those who reported that they did not take photographs for education or research purposes (*n* = 35, Figure 6C) had policies that did not allow photographs of donors (*n* = 13, 37.14%) or required permission from the donor program (*n* = 13, 37.14%). Policies that had less restrictions of (i.e., no‐identifiable features [*n* = 4, 11.43%] and no limitations on photographs [*n* = 2, 5.71%]) were less likely to be used when photographs were not used for educational or research purposes. Fischer's exact test revealed a significant difference (*p* = 0.0002) between the policy and the use for photographs. This suggested that those who have more restrictive policies are less likely to take photographs of human body donors for educational or research use. Additionally, those who reported their policy preventing photographs of donors abided by those policies.

**FIGURE 6:**
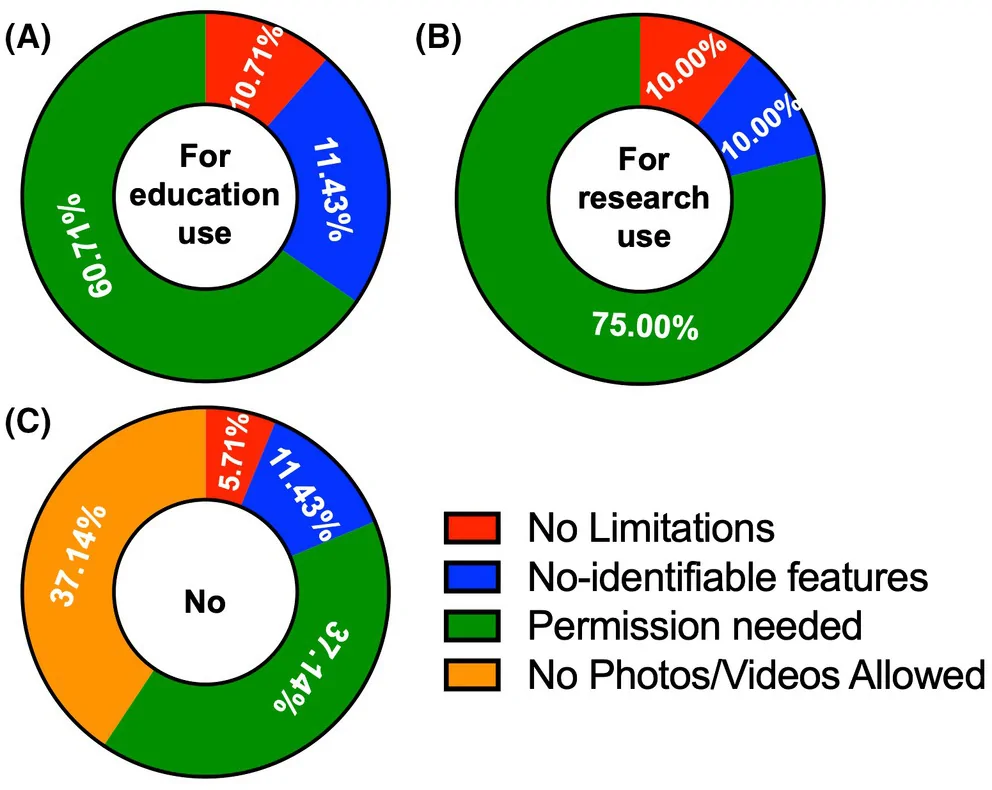
Fischer's exact test analysis of those who take photographs of donors and the policies in place at the institution. (A) Photographs for education use (*n* = 28): No limitations 10.71% (*n* = 3), No‐identifiable features 21.43% (*n* = 6), Permission needed 60.71% (*n* = 17), No photographs/videos allowed 0.00% (*n* = 0). (B) Photographs for research use (*n* = 20): No limitations 10.00% (*n* = 2), No‐identifiable features 10.00% (*n* = 2), Permission needed 75.00% (*n* = 15), No photographs/videos allowed 0.00% (*n* = 0). (C) Do not take photographs of donors (*n* = 35): No limitations 5.71% (*n* = 2), No‐identifiable features 11.43% (*n* = 4), Permission needed 37.14% (*n* = 13), No photographs/videos allowed 37.14% (*n* = 13). Responses of “Other” were removed. No Limitations = “I am able to take any photos/videos I want; there are no limitations”; No‐identifiable features = “I am able to take photos/videos without permission, but with certain regulations (e.g., no exposed faces or identifiable tattoos)”; Permission needed = “I have to request permission to take photos/videos and follow regulations set by the donor program”; and No photographs/videos allowed = “I am not allowed to take photos/videos of donors.” Fischer's exact test on number of occurrences p*** = 0.0002.

This theme continues with those who take videos of human body donors. Overall, the number of respondents who take videos of donors (*n* = 22) is reduced compared to those who take photographs (*n* = 48). When asked about taking videos for educational use (*n* = 18, Figure 7A), the most common policy reported was no videos allowed (*n* = 12, 54.55%), followed by permission required (*n* = 7, 31.82%). For those who take videos for research use (*n* = 4, Figure 7B), all reported a policy where permission was required (*n* = 4, 100.00%). Most of the respondents reported that they did not take videos for either education or research use (*n* = 50, Figure 7C). Of those, the most reported policies were permission needed (*n* = 23, 46.00%), followed by no videos allowed (*n* = 14, 28.00%), no‐identifiable feature (*n* = 5, 10.00%), and policies with no limitations on imaging donors (*n* = 3, 6.00%). Fischer's exact test showed a significant difference between the type of policy and the use of videos (*p* = 0.0144). In contrast to those who took photographs, those who had images were more likely to have no video policies.

**FIGURE 7:**
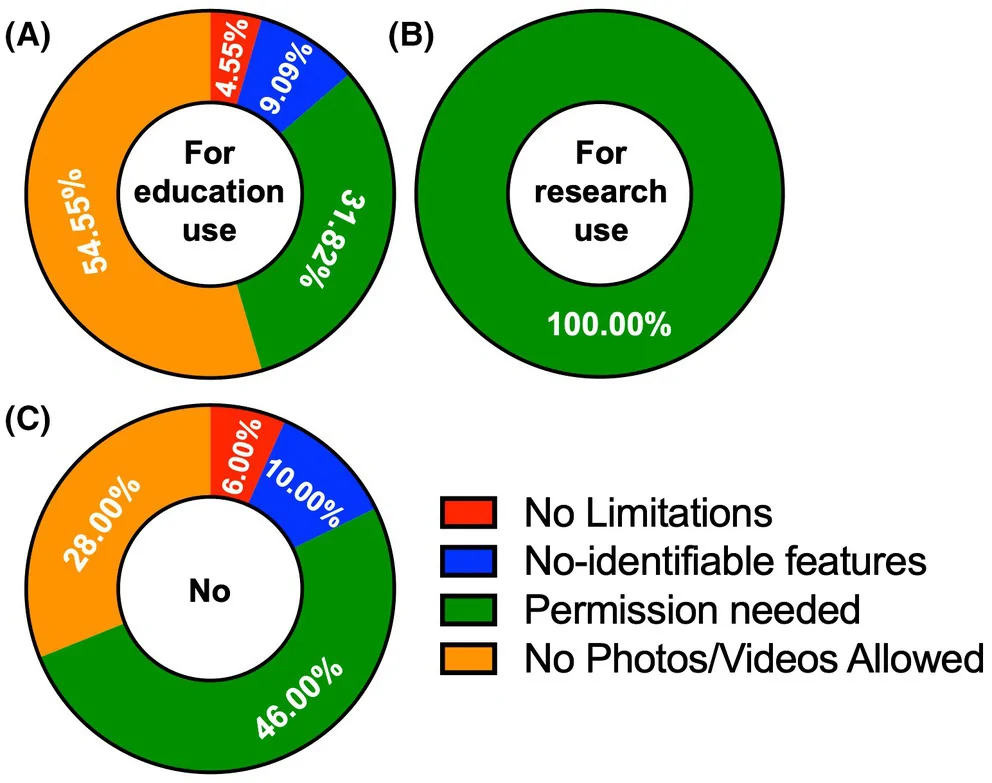
Fischer's exact test analysis of those who take videos of donors and the policies in place at the institution. (A) Videos for education use (*n* = 18): No limitations 4.55% (*n* = 1), No‐identifiable features 9.09% (*n* = 2), Permission needed 31.82% (*n* = 7), No photographs/videos allowed 54.55% (*n* = 12). (B) Videos for research use (*n* = 4): No limitations 0.00% (*n* = 0), No‐identifiable features 0.00% (*n* = 0), Permission needed 100.00% (*n* = 4), No photographs/videos allowed 0.00% (*n* = 0). (C) Do not take photographs of donors (*n* = 50): No limitations 6.00% (*n* = 3), No‐identifiable features 10.00% (*n* = 5), Permission needed 46.00% (*n* = 23), No photographs/videos allowed 28.00% (*n* = 14). Responses of “Other” were removed. No Limitations = “I am able to take any photos/videos I want; there are no limitations”; No‐identifiable features = “I am able to take photos/videos without permission, but with certain regulations (e.g., no exposed faces or identifiable tattoos)”; Permission needed = “I have to request permission to take photos/videos and follow regulations set by the donor program”; and No photographs/videos allowed = “I am not allowed to take photos/videos of donors.” Fischer's exact test on number of occurrences p* = 0.0144.

Lastly, comparisons of the imaging policies reported and how images (photographs or videos) were stored were conducted. This survey presented this “select all that applies” question only to those who reported taking photographs and/or videos, regardless of policy. For those who had policies with no imaging limitations (*n* = 5, Figure 8A), most stored images on their work computer (*n* = 3, 60.00%), followed by the cloud (*n* = 2, 40.00%), and personal computer/laptop (*n* = 1, 20.00%). For those who did not need to request permission, but had to exclude identifiable features (*n* = 12, Figure 8B), most reported storing images on their work computer (*n* = 7, 58.33%) or personal phone (*n* = 6, 50.00%), followed by cloud storage (*n* = 5, 41.67%), personal computers/laptops (*n* = 4, 33.33%), and secure/encrypted server (*n* = 3, 25.00). For those who had policies that required permission to take images of the human body donors (*n* = 36, Figure 8C), most reported storing images on their work computer (*n* = 18, 50.00%) or on a secure/encrypted server (*n* = 18, 50.00%), followed by cloud storage options (*n* = 9, 25.00%). Interestingly, more than 50% of those who took videos for education use reported policies that did not allow photographs/videos.

**FIGURE 8:**
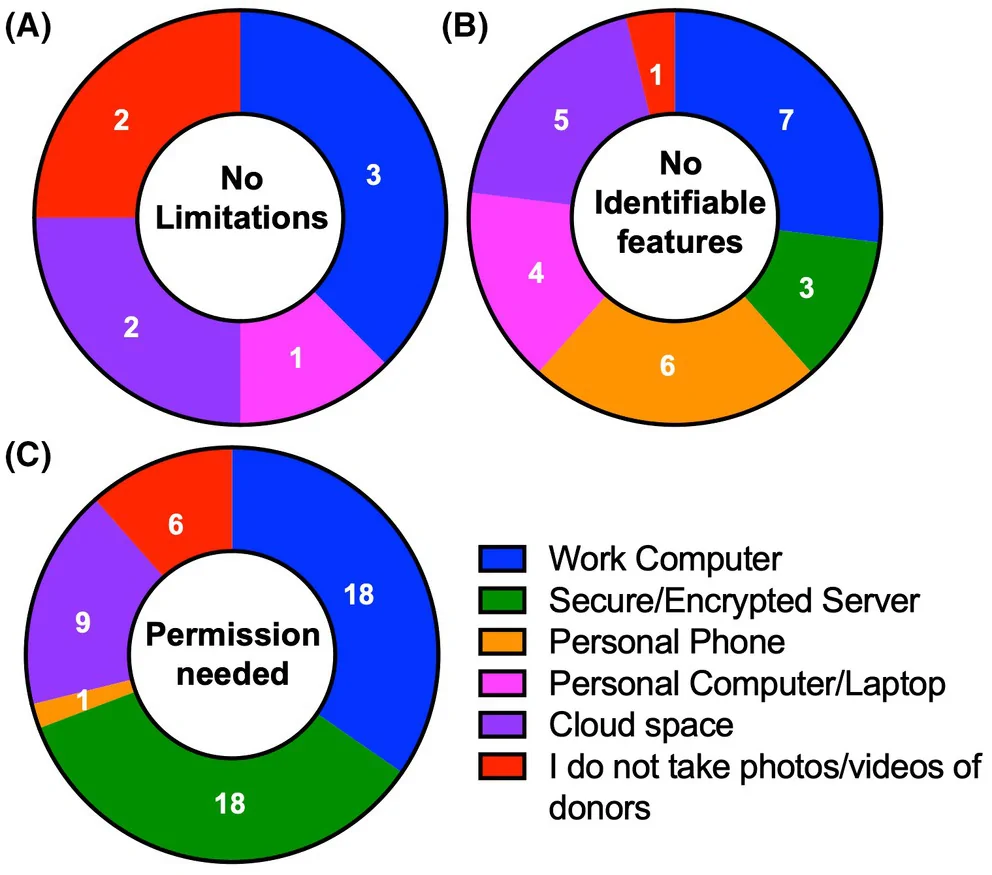
Fischer's exact test analysis of institutional policies and storage for photographs/videos. (A) Policy of no limitations (*n* = 5): Work computer *n* = 3, Secure/encrypted server *n* = 0, Personal phone *n* = 0, Personal computer/laptop *n* = 1, Cloud space *n* = 2, I do not take photographs/videos of donors *n* = 2. (B) Policy of no‐identifiable features” (*n* = 12): Work computer *n* = 7, Secure/encrypted server *n* = 3, Personal phone *n* = 6, Personal computer/laptop *n* = 4, Cloud space *n* = 5, I do not take photographs/videos of donors *n* = 1. (C) Policy of permissions needed (*n* = 36): Work computer *n* = 18, Secure/encrypted server *n* = 18, Personal phone *n* = 1, Personal computer/laptop *n* = 0, Cloud space *n* = 9, I do not take photographs/videos of donors *n* = 6. This questions was select all that apply and the data are reported as number of occurrences. No Limitations = “I am able to take any photos/videos I want; there are no limitations”; No‐identifiable features = “I am able to take photos/videos without permission, but with certain regulations (e.g., no exposed faces or identifiable tattoos)”; Permission needed = “I have to request permission to take photos/videos and follow regulations set by the donor program.” Fischer's exact test on number of occurrences p** = 0.0010.

## DISCUSSION

Since 2011, there has been an active call among anatomists to institute policies to regulate the use of human body donors.^14^ In the last several years, three highly regarded professional societies, the AAA,^22^ the American Association for Clinical Anatomists^17^ and the International Federation of Association of Anatomists,^19^ have published guidelines that provide strong recommendations and guidelines specifically for the imaging practices of human body donors. This study reveals that even with professional society support and published guidelines, there is still no consistent adaptation from academic institutions to implement and/or abide by these guidelines. Fundamentally, there is still considerable work to be done to protect the identity and trust of our donors and their families.

### Current policies and implementation

This study found that there is a significant lack of consistency in the policies driving the imaging of human body donors. Two of the most common policies reported were to (1) keep identifiable features hidden without needing to request permission, and (2) request permission any time an image was to be taken. Comparatively, these policies differ significantly from one another, and each comes with its own risks. For the first policy, by allowing imaging without individual permission, there is a large possibility that records of the images taken do not exist. Tracking of their identity and consent to these practices can become lost. For the second policy, while records will be created, permission can either be granted from an internal or external program. This complicates recordkeeping and adherence to all applicable program or committee policies and opens up unnecessary room for error.

Investigations into consent documentation, particularly those with imaging policies, have seen an increase in recent interest among published manuscripts. Recent studies by Johnson and Zealley revealed that almost 90% of all consent documentation collected for their studies did not mention imaging in any manner.^23^, ^24^ In a survey of Body Donor Program Directors, it was found that approximately 62% of policies required permission before imaging was taken, compared with 50% in this study; approximately 27% required no permission compared with 21% in this study; and 10% were not allowed to take any images compared with almost 20% in this study.^25^ The difference between the two studies on policies prohibiting taking images may be due to directors being surveyed, compared with faculty in this survey. Several respondents mentioned personal ethical and moral beliefs that were in conflict with the policies they were instructed to follow, which might explain these differences.

Dissecting a little deeper, differences in policies are more pronounced when looking at where the donors are procured and how many are being used. Programs that used more than 40 donors were more likely to be from internal programs, whereas programs that used less than 40 donors were more likely to source from external body donation programs. External body donor programs were more likely to have an even split of policies, but showed a higher instance of policies where no photographs were allowed, compared with the larger, internal programs. Comparably, while internal programs were more likely to need requested permission before taking any imaging, they were also much more likely to not have any policies for imaging at all. External programs that do not allow photographs are likely attributed to the distribution of donors to not only multiple institutions, but also potentially out of the state they are based in. The US Revised Uniform Anatomical Gift Act (UAGA) of 2006 worked to smooth out inconsistencies between states' policies surrounding anatomical gifts, largely focused on (but not limited to) organ donation.^26^ However, shortly thereafter, many states adopted amendments or provisions to this Act, and some failed to ratify the updated Act at all,^27^ which may inspire uncertainty about what might be legal in the states where external BDPs are sending donors. By prohibiting imaging outright, external body donor programs remove the need for monitoring and verifying a patchwork of different states' policies. On the other hand, internal programs, including Anatomical Oversight Committees, are poised to handle multiple requests within a finite geographic reach, thus the establishment of permission before any imaging can occur.

Even though most internal programs are better positioned to implement and uphold any new policies, this study found that they are also more likely to not have any limitations on imaging the donors. After all, lack of a policy does not equal prohibition. The question becomes, is this true that several institutions do not have any policies regulating imaging donors, or is this a result of the respondent not knowing/understanding the policy at their institution? To the latter question, more than 15% of respondents indicated that they did not take images because they did not know the policies in place at their institution. This illuminates a major concern: Even with policies in place, they are rendered moot if those who are meant to follow them do not know of their existence.

Interestingly, this influence on policies was independent of the type of institution and how the donors were used (i.e., prosection, dissection, and research) within their respective schools, but was dependent on the type of image taken, photographs or videos. Photographs were two times more likely to be taken of donors compared with videos, with education use being more likely than for research use. Policies that prevent imaging were more likely to be upheld in regard to photographs compared with videos. Interestingly, for those who took videos for educational use, the most common policy reported was “no photos/videos allowed.” This dichotomy reveals a potential disconnect between photographs and videos and how faculty and/or policies perceive photographs vs. videos. Policies could be specifying “imaging” as a general term to encompass photographs, videos, and radiologic imaging, or policies could only mention photographs. Additionally, if photographs and videos are grouped under general terms such as imaging, interpretation of this term could be left to the educator to determine the definition.

### Privacy and security concerns

The shielding of the privacy and security of our donors and their families is paramount to ensure continued relationships in the future. IFAA guidelines state that any and all digital images must be stored on secure devices.^19^ This study found that more restrictive policies, particularly those that required permission to take images, were more likely to have educators storing those images in more secure locations, such as a work computer or secure/encrypted server. As the policies become less restrictive (i.e., not requiring permission) the more likely images are stored on personal devices, such as personal phones and/or computers. This suggests that the policies are working when imaging of donors is limited.

However, storage of media containing donor tissues on personal devices is of increasing concern due to the wide access of cameras on personal devices. Faculty often use their phones in the laboratory to teach (i.e., bring up diagrams and pull up lecture slides), to take attendance, to look things up, and for other educational actions. Additionally, while student personal devices, such as phones and tablets, are traditionally prohibited in anatomy laboratories, it is becoming increasingly common for students to use digital devices to access anatomical structure lists, dissection guides, presentation slides, etc. While some universities provide students with these devices, many others utilize their own devices.

Even with policies in place for proper image storage, any authorized or unauthorized photograph or video is at significant risk of being posted to outside, publicly accessible sources. This is particularly worrisome as the use of social media, including content that is centered around anatomical educational, has only increased.^28^ One of the first accounts of donor images being posted to social media occurred around 2010, when a Stony Brook University Medical Center student posted a selfie with a donor on Facebook.^29^ Since then, hashtags have been used (e.g., #medlife) was used to post donor selfies as an example of a “day in their life” content.^30^ On another token, full accounts are dedicated to showing educational content on human body donors and their tissues, such as @instituteofhumananatomy on TikTok.

Despite the focus on education, there remains a notable lack of transparency into the consent process for this type of imaging. Current consent language generally lacks specific policies that limit the posting of human tissues, with or without imaging policies.^23^, ^25^ Problematic aspects of posting human tissues are compounded by the added layer of for‐profit motive, with courses, merchandize, etc. available for purchase. The combination of profit motive and lack of consent has led to exploitation of human body donors. This recently occurred in an Oregon Hotel ballroom in 2021 where a live dissection was performed by a retired faculty member from Montana University.^31^ This public dissection was done without the consent and/or knowledge of the public use and distribution of the video online. The family thought they had entrusted their loved one to a non‐for‐profit entity, but were not informed on the sale of their loved one and felt that the organization had not upheld their promises and had broken their trust.

Trust is a key factor in donors and families deciding to donate.^25^, ^32^, ^33^ The mistrust donors and families have of body donation for education has continued into current times and has significant consequences for body donation and medical education when broken. Minority populations, many of whom have been medically victimized through history, report significant distrust in the donation process and are greatly underrepresented in human anatomy donor populations. Research on the matter shows that among these populations, there is a substantial lack of trust that medical schools, even in more modern times, will respect their remains.^34^ The consequences of broken trust are well illustrated by the decrease in registrations and the withdrawal of over 60 registrants in 2024 after the conviction of the Harvard Medical School morgue manager and in the wake of the subsequent lawsuits.^35^, ^36^

### Balancing opinions

While there is generally strong support within anatomical education for policies that limit the imaging of human body donors, this is not universally well‐received. Following the IFAA Recommendations for Good Practice Around Human Tissue Acquisition and Use in Anatomy Education Research, a counter commentary was published by J. Iwagana and 34 other authors and editors that described the damage such policies could induce on anatomy laboratories, particularly on textbooks and other educational material with the regulations of donor images for commercialization.^37^ This disagreement is echoed in this study as multiple respondents noted actions that differed from policies at their institutions. Their personal ideology was evident in comments made within the free response section. An example of two of their comments is below:

We choose to take no pictures out of respect.

I do not take photos of the donor, but I believe it is allowed under a narrow set of conditions.

While these actions were more strict in contradiction to policy, it cannot be ignored that there will also be those who will act more leniently to their directive policy.

On the contrary, changes in terminology have had a significant effect on some educators. Notably, one respondent focused almost all of their responses in the survey on the term “human body donor” in favor of the outdated and objectifying term, “cadaver,” refusing to answer several survey questions. Regardless of why there is a contrasting opinion, dissenting voices will always occur with any changes made, but their views and concerns cannot be ignored, and instead, need to be addressed for compliance.

### Action steps

As the HAPS ADS Committee, we recognize that one of the challenges is the standardization and enforcement of these policies. If academic institutions and body bequeathal programs do not institute and practice policies, such as those listed above, there is an elevated risk of future issues that could affect the trust individuals have with donating their body. Below are action steps that can be taken to start the standardization process:

- *Uniform Imaging Policies*: Body donation programs should consider adopting standardized policies for imaging practices, such as no images/videos within the laboratories without the explicit consent of donors and images/videos taken can only be stored on secure, institutional servers (i.e., not on a flash drive).
- *Transparency is Key*: It is important to ensure that registered donors and the family members of the donors have a correct and complete understanding of what their loved one's gift is being utilized for. While the extent of individual dissection does not need to be discussed, information about the policies regarding the care and respect for the donors, including imaging policies, should be clearly available. Unfortunately, there have been disturbing stories, both historically and in recent times, regarding anatomical dissections and inappropriate handling of human body donors. As such, being transparent about what is allowed and not allowed for each program, and demonstrating that these policies are being enforced, is important in building trust with donors and their families.
- *Special Consent for Specific Imaging Practices*: When an individual donates their body, depending on the program's consent form and the varying policies across programs, specific and clear mention of imaging policies should be noted. Care should be taken to not overwhelm donors with consent language as this can negatively impact their perception and action in donation.^25^ As such, the ADS Committee recommends a clear and concise consent form regarding what is allowed or not allowed in terms of imaging. This should relate to imaging and videos for teaching or research purposes (i.e., taken by faculty), those used for various outreach programs, and additional radiology imaging (e.g., CT scanning).
- *Ethical Discussions with Students*: A critical conversation to have with all students who learn from willed body donors is about ethics. These conversations should underscore the importance of proper care of the donors and discussions of ethically focused laboratory policies (e.g., no one in the laboratory except for registered students, no photographs). These conversations could also turn into larger discussions or assignments, such as student opinions on the usage of anatomical donors to educate the public via social media platforms (e.g., TikTok).
- *Remind Students that Body Donations Include More than Dissections*: Many students will actively remember policies regarding proper care of donors for full‐body donors and large prosections, but students should be regularly reminded that other materials in the classroom are from willed body donors, such as plastinated prosections and bones. The policies for these materials should be consistent with those of whole‐body donors.
- *Update Policies Regularly*: Policies should be regularly reviewed by a designated person or committee within body donor policies as updates and changes to policies can occur at the federal, state, and/or institutional level. Policies may change due to a variety of reasons, and these policies should be clearly communicated to all involved parties. Evolution of these policies can occur at the federal and/or institutional level. Additionally, consistent review of emerging technologies should be considered for policy updates, such as banning certain eyewear in the anatomy laboratory, including those with artificial intelligence capabilities and those that contain cameras. This should include clear instruction to faculty, staff, and students on the expectation to uphold the policies.
- *Lead by Example*: Faculty, administrators, laboratory managers, and even teaching assistants should lead by example in how they care for the donors. This care ranges from proper storage of the donors, to terminology (e.g., not using the term “cadaver”), and not taking unauthorized images—including as part of official institutional marketing.

All of these steps are not possible, first, without the input and effort by those who work with human body donors and the collaboration of different organizations, including the AAA, HAPS, and the IFAA. Without their collective actions, changes to policies outside of the institutional level will continue to be individual as those at the state and federal level are not aware of challenges and ethical considerations that are experienced daily.

## LIMITATIONS

This study did have some limitations. Compared with the amount of human body donor programs, the sample size was small. While there was some even distribution among the categories, some groups were overrepresented where the small sample size and the non‐even distribution could potentially skew the data. Lastly, while this study did investigate imaging policies from an educator's perspective, separation between who was allowed to take images of human body donors (students vs. faculty) was not specified. Previous students have shown that some policies allow for student images; however, this is more than two‐thirds less than those that allow faculty to take images.^25^, ^38^

## CONCLUSION

This study exposes the variety of imaging policies and the actions of those in gross anatomy laboratories in contrast to guidelines and recommendations from professional societies. While there were not clear factors that contributed to the type of imaging policies, the number of donors and where human body donors were supplied did influence the policies used. Importantly, while there is an ongoing effort to limit the imaging of human body donors and protect their privacy with secure storage of those images, there is still significant progress to be made. Without significant effort from everyone who works with human body donors, the lack of uniform policies will continue.

## AUTHOR CONTRIBUTIONS

**Bobbie J. Leeper:** Conceptualization; investigation; writing – review and editing. **Kelsey E. Stevens:** Conceptualization; investigation; writing – review and editing. **Rhiannon E. Robinson:** Conceptualization; investigation; writing – review and editing. **Danielle N. Edwards:** Conceptualization; investigation; writing – original draft; methodology; writing – review and editing; formal analysis. **Jeremy J. Grachan:** Conceptualization; investigation; writing – review and editing.

## CONFLICT OF INTEREST STATEMENT

The authors have no conflicts of interest to report.

## ETHICS STATEMENT

The survey in this study was deemed exempt by the University of Alabama at Birmingham Internal Review Board (IRB‐300012853).

## Data Availability

The data that support the findings of this study are available from the corresponding author upon reasonable request.
